# An Early Detection Circuit for Endurance Enhancement of Backfilled Contact Resistive Random Access Memory Array

**DOI:** 10.1186/s11671-021-03569-0

**Published:** 2021-07-05

**Authors:** Yun-Feng Kao, Jiaw-Ren Shih, Chrong Jung Lin, Ya-Chin King

**Affiliations:** Microelectronics Laboratory, Institute of Electronics Engineering, National Tsing Hua University, Hsinchu, 300 Taiwan

**Keywords:** RRAM, Variability, Stochastic model, Monte Carlo Simulation, Trap-assisted tunneling

## Abstract

As one of the most promising embedded non-volatile storage solutions for advanced CMOS modules, resistive random access memory’s (RRAM) applications depend highly on its cyclability. Through detailed analysis, links have been found between noise types, filament configurations and the occurrence of reset failure during cycling test. In addition, a recovery treatment is demonstrated to restore the cyclability of RRAM. An early detection circuit for vulnerable cells in an array is also proposed for further improving the overall endurance of an RRAM array. Lifetime of RRAM can be extended to over 10 k cycles without fail bits in an array.

## Introduction

In recent years, RRAM with advantages of simple structure, superior scalability and high compatibility to advanced CMOS processes has become one of the core technologies for realizing embedded non-volatile memory modules [1–8]. RRAM featuring high cyclability can extend its applications to systems which required to update non-volatile data more frequently, such as computing-in-memory and neuromorphic systems [9–15].

Switching of states in RRAM films is believed to be attained by generation/recombination of oxygen vacancies (*V*_o_) to further control construction/destruction of conductive filaments (CF) [16–22]. Many studies have shown that stochastic mechanisms in the formation of CFs during set/reset operations have been found as one of root causes for bit failure during cycling tests [23–27]. In a fail-to-set cell, excess *V*_o_ recombined during reset operations widens the tunneling gap between the residual CFs and the top electrode, which weakens the electric field in the gap region, leading to low *V*_o_ generation [24]. On the other hand, excess *V*_o_ generated during set operation, leading to CF over-growth. This is believed to be the main cause of reset failures [24, 28]. Also, unexpected depletion of oxygen ions during cycling is found to be responsible for the closing of the resistance window [23, 28]. Several schemes for mitigating the effect of the stochastic process in *V*_o_ generation/annihilation have been reported in various studies [23, 24, 28–30]. Pulse with large rising and falling time was found to, respectively, reduce *V*_o_ generation in set operation and consumption of oxygen ion in reset process [23]. To obtain good controllability of *V*_o_, Chen *et al*. also suggest a tuning-pulse amplitude method for balancing set/reset operations [29]. Aside from pulse conditioning [23, 29], strong set/reset electrical recovery treatments on devices after endurance failure are found that cells can be restored and cycled again [24, 28, 30]. It is also revealed that increased frequency of recovery operations pushes the overall cycling endurance performance higher [24]. Periodic recovery on the whole array during cycling costs high power overhead and implementation challenges in real memory modules. Therefore, find the weak cells, which are at the brink of cycle failure, is essential to the implementation of a selective and timely recovery. This can enable the improvement of cyclability without wasting unnecessary treatments on healthy cells.

In our previous work, cells with low reset efficiency were found to correlate to its CF topography. Furthermore, random telegraphic noises are linked to the CF types, which also reflect the change of CF after cycling stress [31]. In this work, new circuits for early detection of weak devices in an array by its read current characteristics have been proposed. A reset recovery operation is also introduced for preventative strengthening of vulnerable cells identified by the detection method. Applying the method of early detection and selective CF strengthen operations, significant improvement in the set/reset cyclability has been successfully demonstrated.

## Methods

**Fig. 1:**
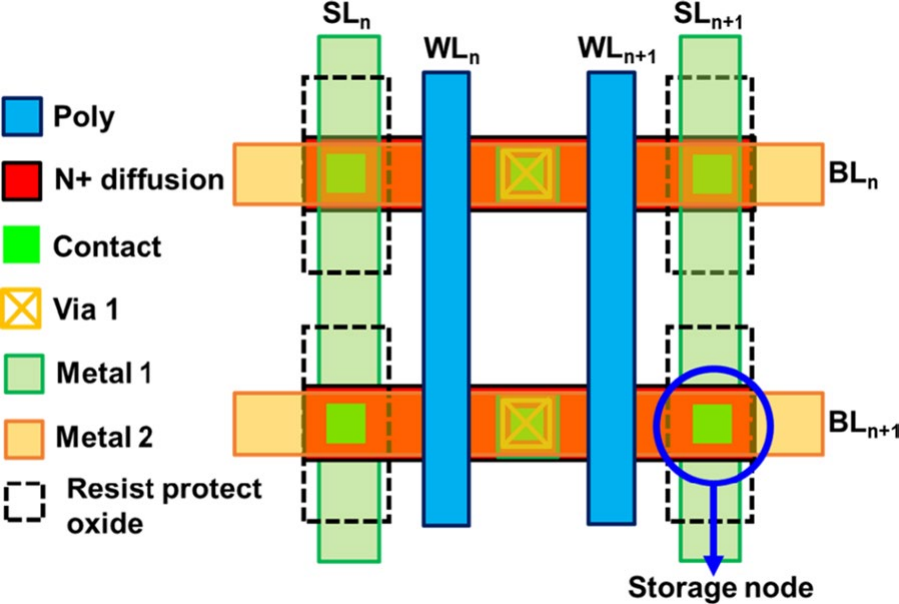
The layout arrangement of the 16 × 16 NOR type backfilled CRRAM array sample under investigation

**Fig. 2:**
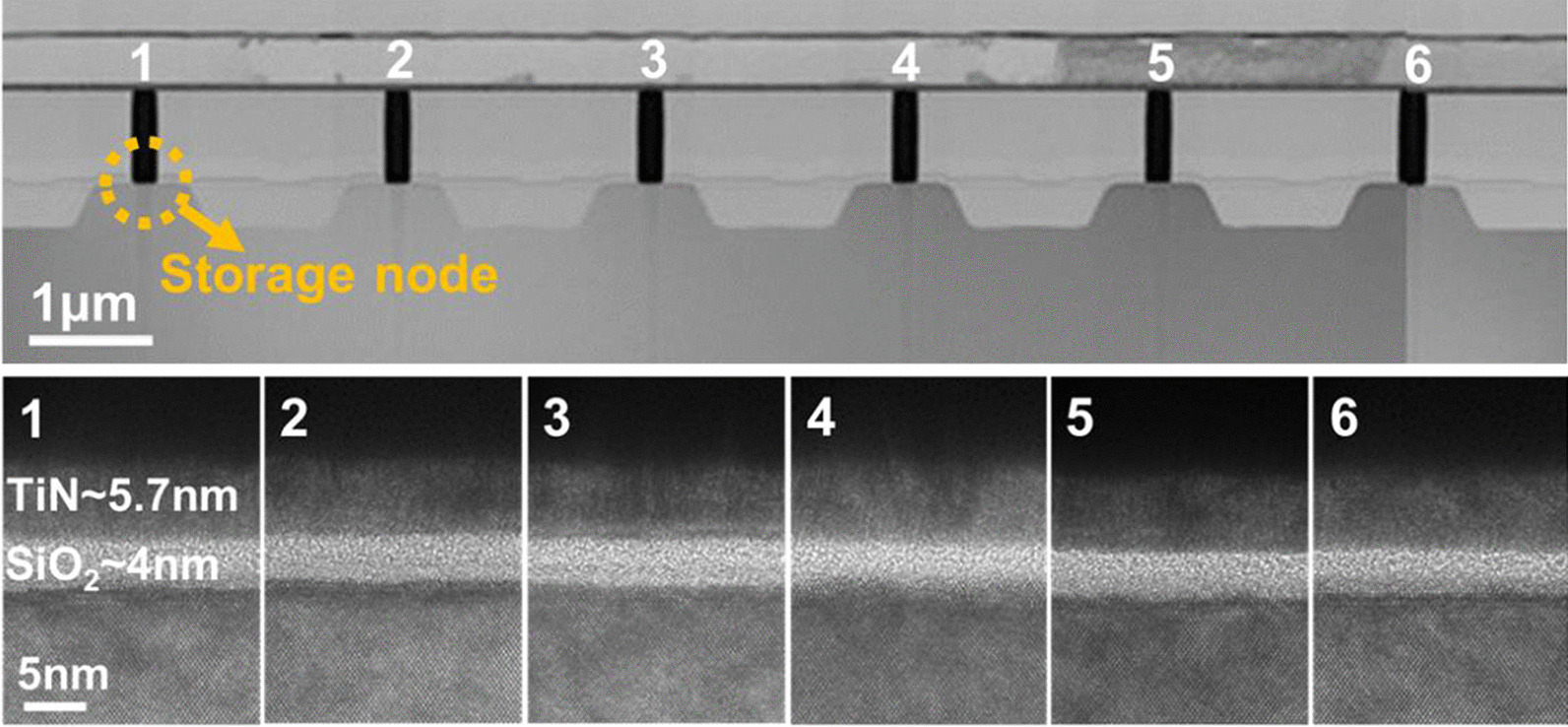
Cross-sectional TEM images of BCRRAM array and cells. Uniform dielectric thickness can be obtained in BCRRAM cells

**Fig. 3:**
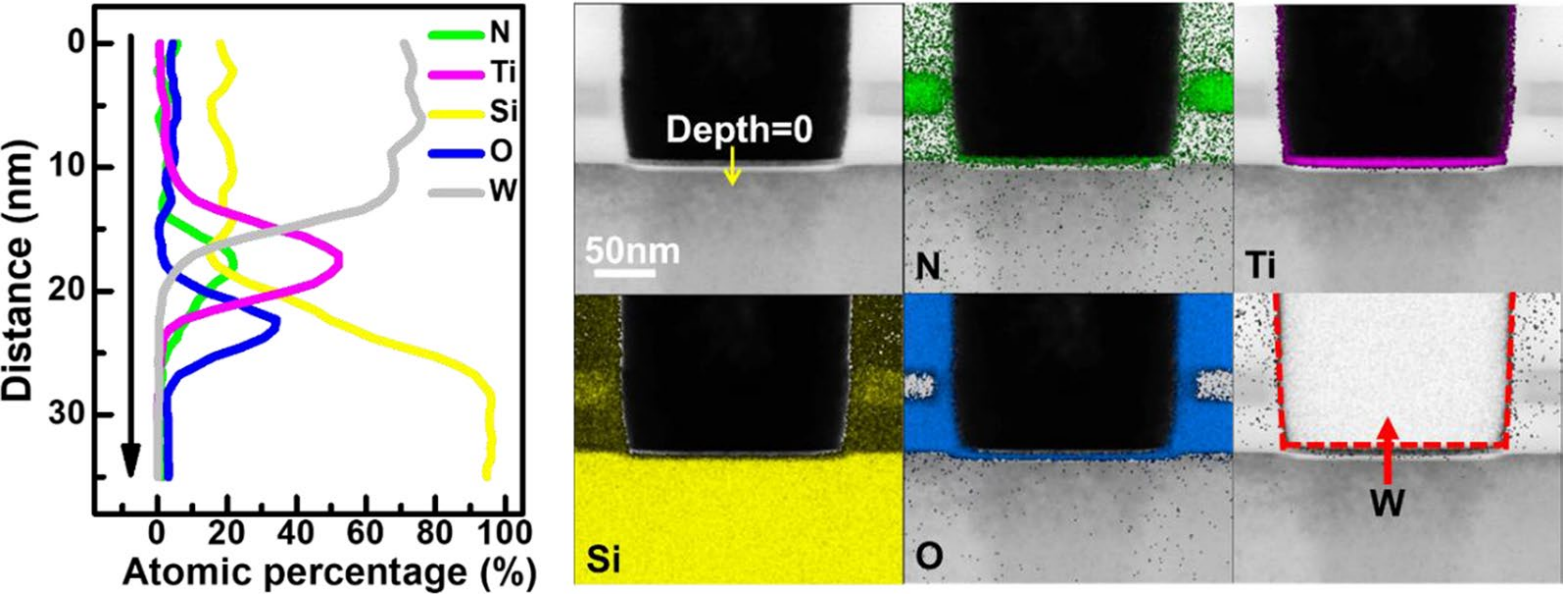
EDX-based composition analysis of TMO layer of BCRRAM cell. BCRRAM’s TMO layer is composed of TiN/TiON/SiO_2_ stack

**Fig. 4:**
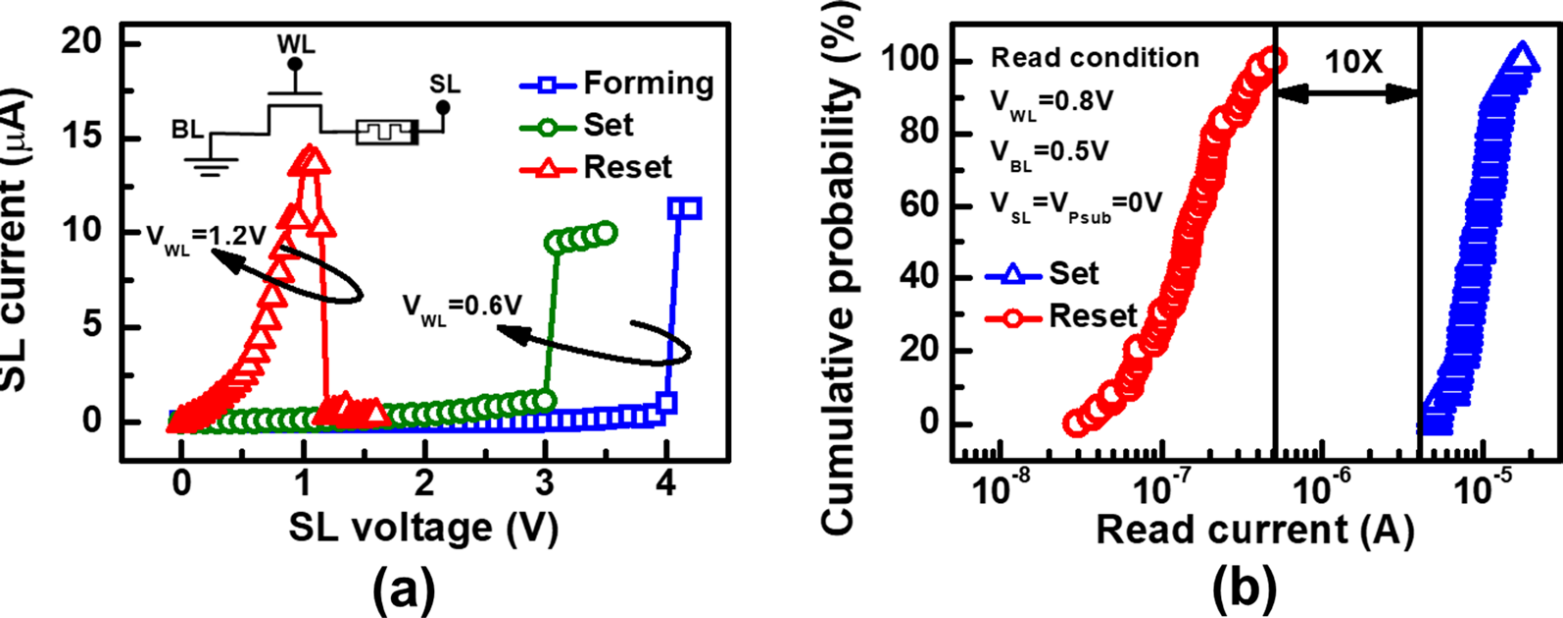
**a** DC forming/set/reset sweep characteristics, which bit lines (BL), are grounded. **b** Current levels after 50 set/reset operations. LRS/HRS are defined as 5 μA/0.5 μA, respectively, to obtain 10 × on/off ratio

Statistical analysis on endurance of RRAM is collected from a 16 × 16 backfilled contact resistive random access memory (BCRRAM) array, which is fabricated by 0.18 μm CMOS logic process [32, 33]. As shown on layout in Fig. 1, storage node of BCRRAM is connected in series with a n-channel transistor for cell selection in a NOR-type array. To in-depth investigate the physical properties of TMO layer of BCCRAM, the transmission electron microscopic (TEM) analysis is carried out by JEOL JEM-2800 transmission electron microscopic with energy 200 keV. The cross-sectional TEM images of BCRRAM array along source line (SL) direction are shown in Fig. 2. Because of the better control of the backfilled dielectric film thickness, uniform transition metal oxide (TMO) layer can be achieved. The relative element compositions along the depth of RRAM film are provided in energy-dispersive X-ray (EDX) spectroscopy analysis in Fig. 3, where TMO film of BCRAM cell is found to be composed of TiN/TiON/SiO_2_ [32, 33]. The electrical analysis is completed by a semiconductor parameter analyzer and a pulse generator. DC forming/set/reset characteristics of BCRRAM are demonstrated in Fig. 4a. Note that high SL voltage in forming/set operations are required to trigger the soft breakdown process. Low word line (WL) voltage 0.6 V on the gate of the select transistor clamps the surge current and prevents to overset into irreversible resistance states. As a BCRRAM device under the unipolar mode, higher *V*_WL_ of 1.2 V is chosen to supply high enough current to enhance the diffusion of oxygen ion and recombination with *V*_o_, favoring the switching back to HRS [34–37]. As shown in Fig. 4b, 10 × read current window can be maintained under 50 DC set/reset cycles, which low resistance state (LRS)/high resistance state (HRS) are set to 5 μA/0.5 μA, respectively.

## Results and Discussion

### Cyclability and Reset Recovery Scheme

**Fig. 5:**
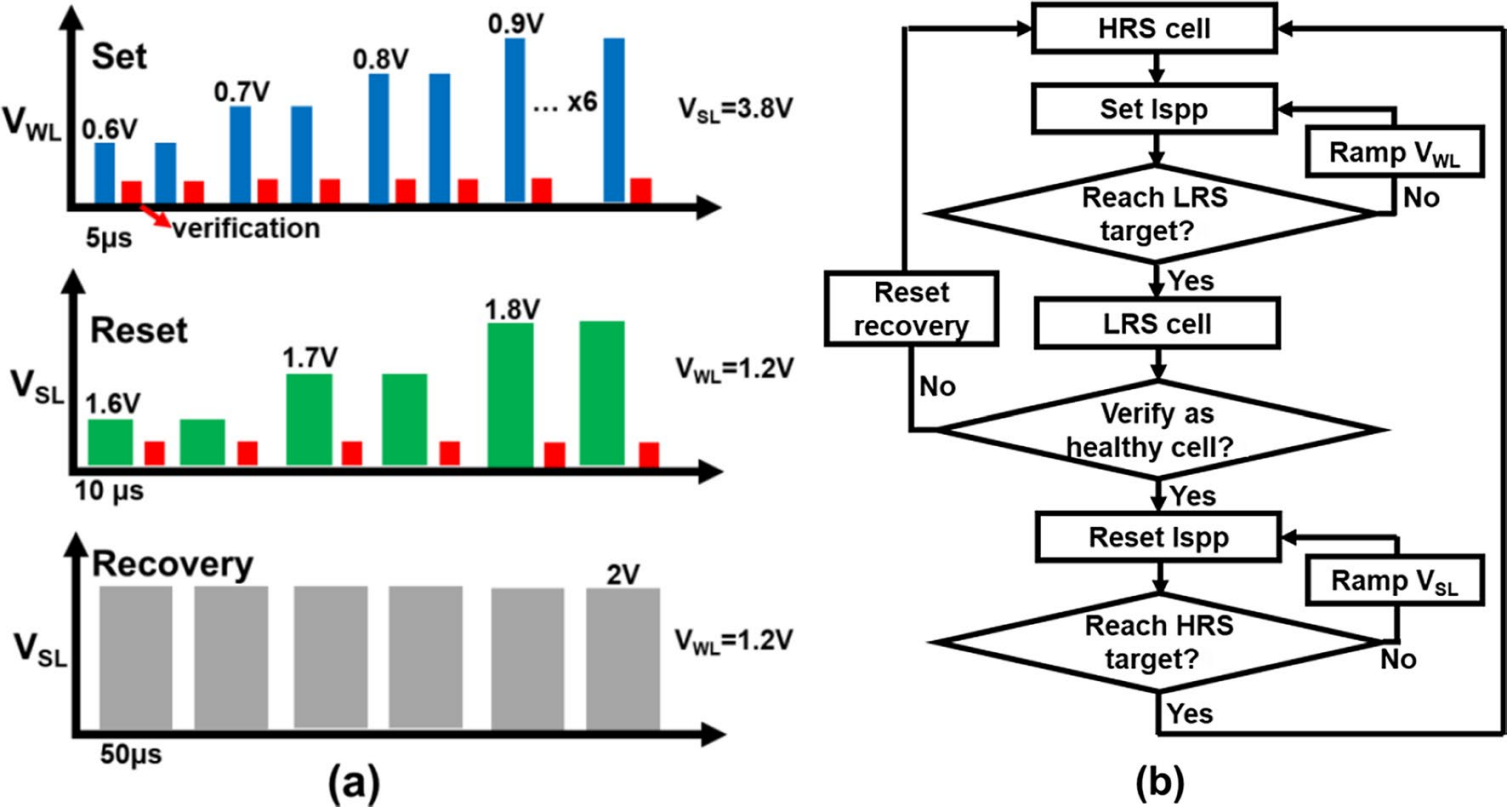
**a** The set/reset ISPP algorithm and recovering reset conditions for cycling tests. The V_WL_ and V_SL_ are ramped, respectively, in set/reset operations. **b** The algorithm for early detecting the weak cells

**Fig. 6:**
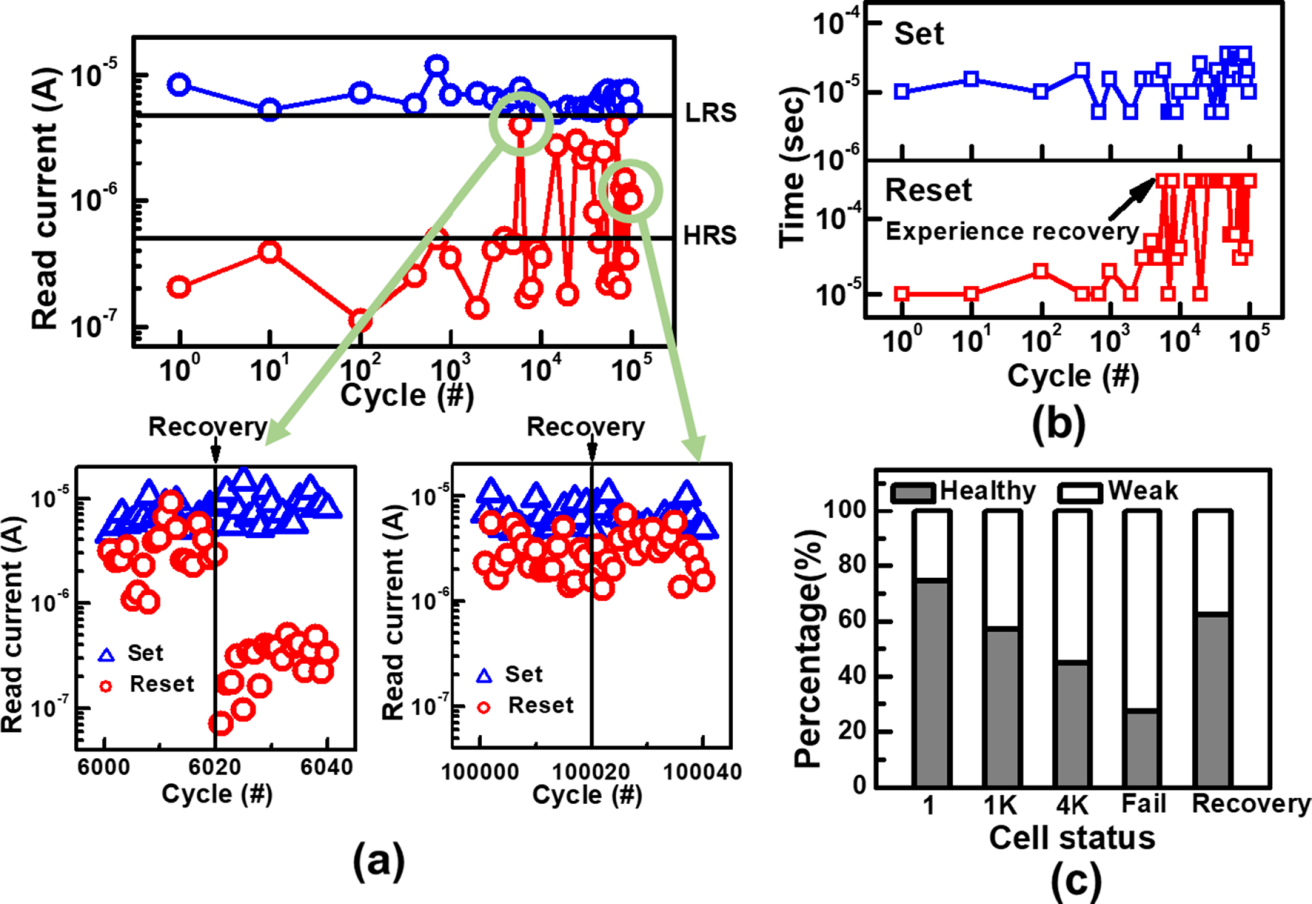
**a** 100 k ISPP set/reset cycles. Cells lost read widows after 6 k cycles can be recovered by 5 strong reset pulse with conditions *V*_WL_ = 1.2 V, *V*_SL_ = 2 V and pulse width of 50 μs. The reset recovery treatment is invalid after 10 k cycles. **b** Set/reset time required to complete state switch during 100 k cycles. **c** Shift in cell type, defined by their noise features, are found during ISPP cycling tests

**Fig. 7:**
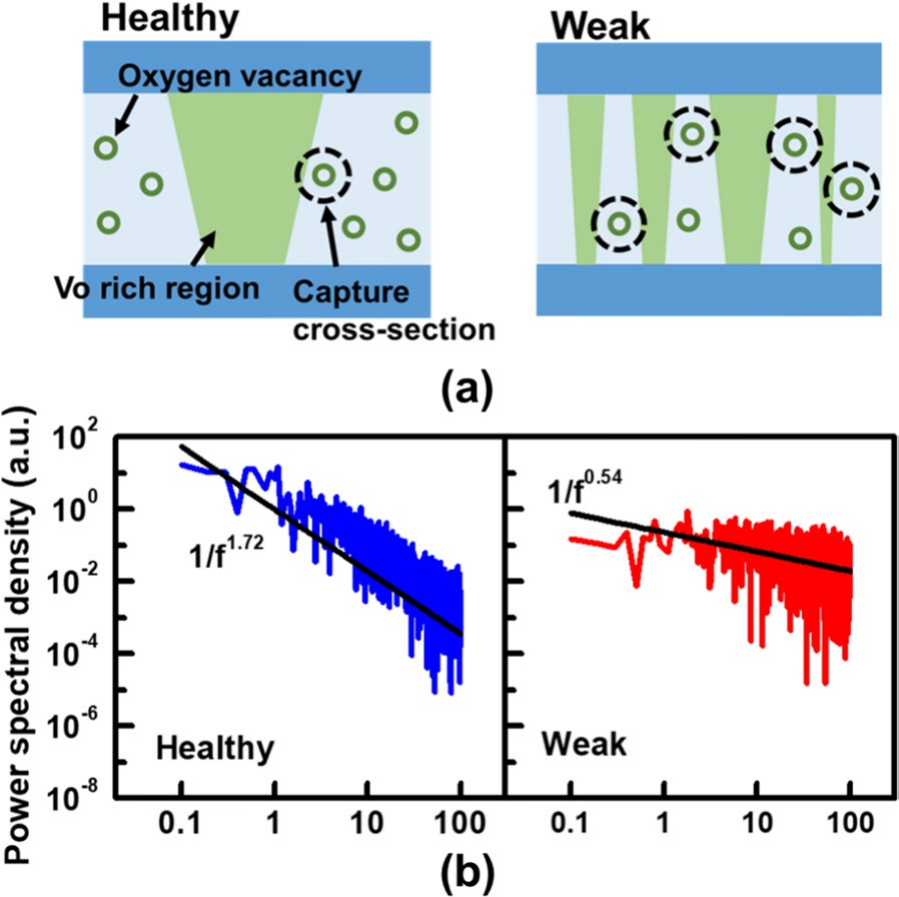
**a** Illustrations of the filaments and trap states on the TMO layer in healthy/weak. **b** Corresponding fitting trends in noise spectrums

Cycling endurance of BCRRAM is examined by an optimized incremental step pulse programming (ISPP) algorithm shown in Fig. 5a. After each stress pulse, the states of BCRRAM are then verified to determine whether *V*_WL_/*V*_SL_ needs to be increased for the next set/reset operations [38]. As displayed in Fig. 6a, stable read current window can be obtained within 20 μs set/reset time, see in Fig. 6b, for 1k cycles. Experimental data show that reset time required to reach the target HRS gradually increases when the number of cycles passes 1000. Data also reveal that most cells eventually stuck at LRS even after raising reset time to 60 μs. To investigate the root cause of reset degradation during cycling, low frequency noise (LFN) found in the read current is investigated and is reported as an index reflecting the properties of CFs [39–41]. In our previous work [31], cells with different densities of CFs inside its TMO layers exhibit distinct noise spectrum in its read current. As depicted in Fig. 7a, cells can be categorized into two groups based on characteristics of the LFN spectrum in their read current, in Fig. 7b. Cells with low density of CFs, labeled “healthy,” are found to be more robust and are expected to endure more cycling stress. Cells containing multiple tiny CFs, named “weak,” are believed to be more vulnerable to stress. To study the main failure mechanisms for cells under cycling tests, LFN of BCRRAM devices are monitored. As summarized in Fig. 6c, a strong correlation between cell types and number of cycles is found in BCRRAM array. Portion of weak cells with multiple conductive paths in TMO layers significantly increases after cycling, which is believed to cause less efficient heating in a dispersed CF, slowing down reset process [31]. As a result, endurance failure on reset operation is attributed to the generation of multiple conductive paths. In addition to ISPP tests, different kinds of CFs generations after the constant voltage stresses were also reported [27, 28]. Unnecessary CFs generated by fixed set/reset operation conditions was believed to be one of reasons, causing cells to gradually lose their capabilities on switching back to HRS. To revive cells after reset failure, unnecessary CFs inside their TMO layers need to be trimmed through strong reset recovery pulses with conditions in Fig. 5a, *V*_WL_=1.2V, *V*_SL_=2V and pulse width of 50 μs, as demonstrated in Fig. 6a. With the proper reset recovery treatment, the read current window as well as its cyclability can be restored. However, as shown in Fig. 6b, the reset recovery pulses are needed more frequently on cells that experienced more than 10 k cycles. Data in Fig. 6a also indicate that reset recovery operations may become useless for some cells after long cycling stress, suggesting that the CFs in these cells are damaged to beyond repair.

The shift in cell’s noise feature observed in Fig. 6c implies that cell types are useful index for repairing a vulnerable cell before it reaches complete failure. Therefore, distinguishing cell types by its noise features during operation is a crucial factor in realizing early interventions for strengthening CFs.

### Early Detection Circuit

For cells with more CFs inside TMO layers, its read current fluctuates between multiple resistance states. On the contrary, cells with one dominant CFs in RRAM films, current repeatedly jump between two distinct states, which can be used as the indices of the healthy cells [31]. As a result, number of middle states in read current can help us identify the vulnerable cells before it fails completely. Therefore, in the algorithm shown in Fig. 5b, to early detect a vulnerable cell and revive it before completely losing its cycling capability, the sampling current of cells is fed to the detection circuit. Once diagnosed restoration operations are performed on the confirmed weak cells. Hence, two circuits for detecting these weak cells are introduced and discussed in the following sections.

**Fig. 8:**
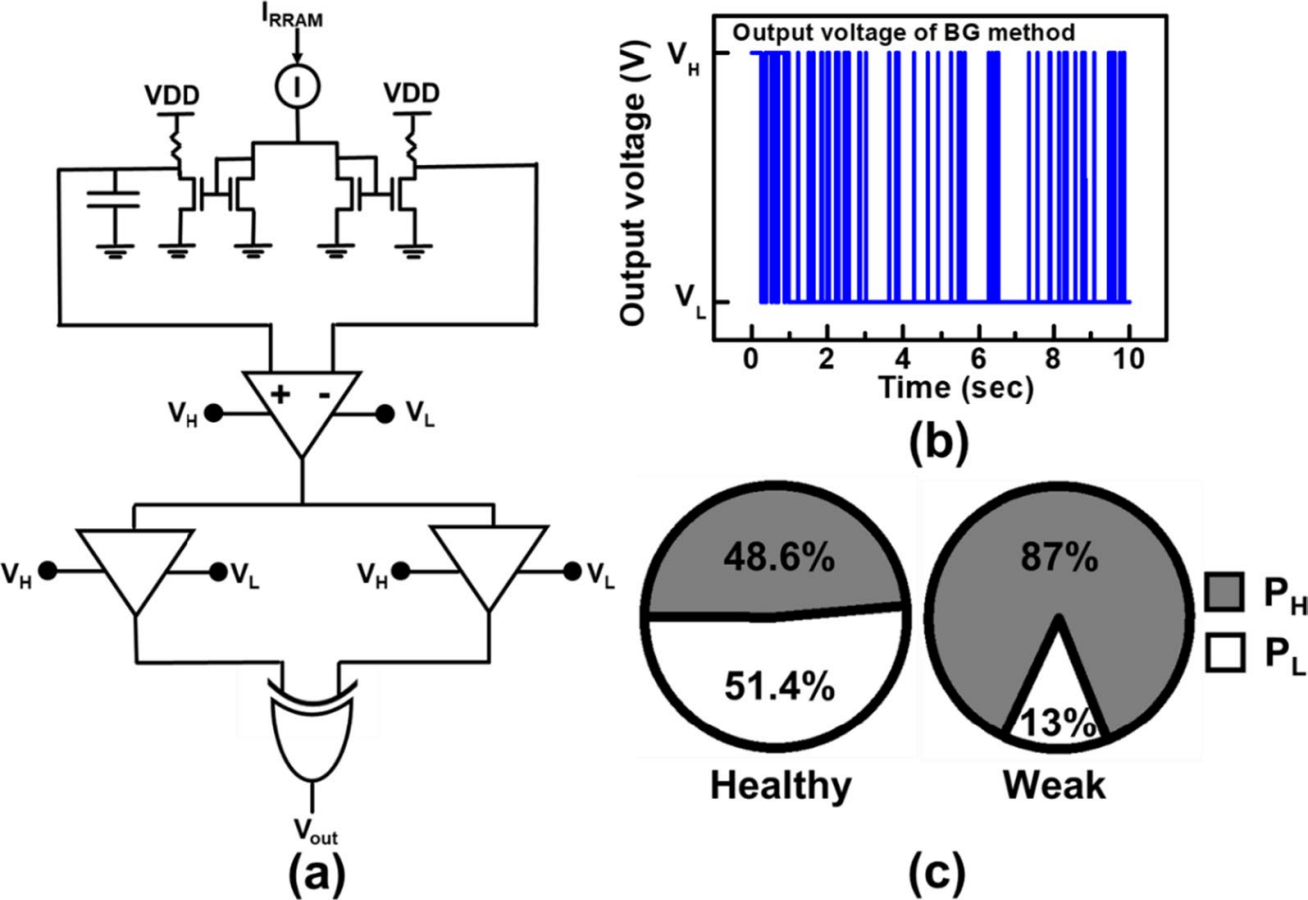
**a** Schematic of BG detection circuit and **b** its voltage output. **c** Pie charts of output voltages on healthy/weak cells in BG method. High portion of high states in a weak cell, which read current rapidly switch between multiple resistance levels

The first detection circuit by the buffer gate (BG) method, is illustrated in Fig. 8a. First, sampling current from BCRRAM cells is mirrored and filtered by a capacitor to set an average level. Next, the difference between the two sides is amplified. The amplified difference of middle states still slightly fluctuates between 0.55 V and 0.45 V. On the other hand, cell with one dominant CF, where the read current is found to jump between bi-levels; when it passes through the detector circuit, the output can be push to the high/low voltage levels. As shown in Fig. 8b, different logic states are generated by the two BGs with proper transition voltages and the XOR logic gate. For cells exhibiting middle-state RTN, the output voltage (*V*_out_) becomes latch at high voltage states (*V*_H_) instead of low voltage states (*V*_L_). The ratio between the probability of output in *V*_H_ (*P*_H_) versus that in *V*_L_ (*P*_L_) of the XOR output on cells first categorized by their LFN as healthy/weak cells are summarized in Fig. 8c. For cells with multiple current levels in read current levels, a larger portion of XOR output remains in high states when the weak cells are put into the detection circuit. On the other hand, healthy cells with a single dominant CF and distinct resistance levels are more likely to put the XOR output at the low voltage states.

**Fig. 9:**
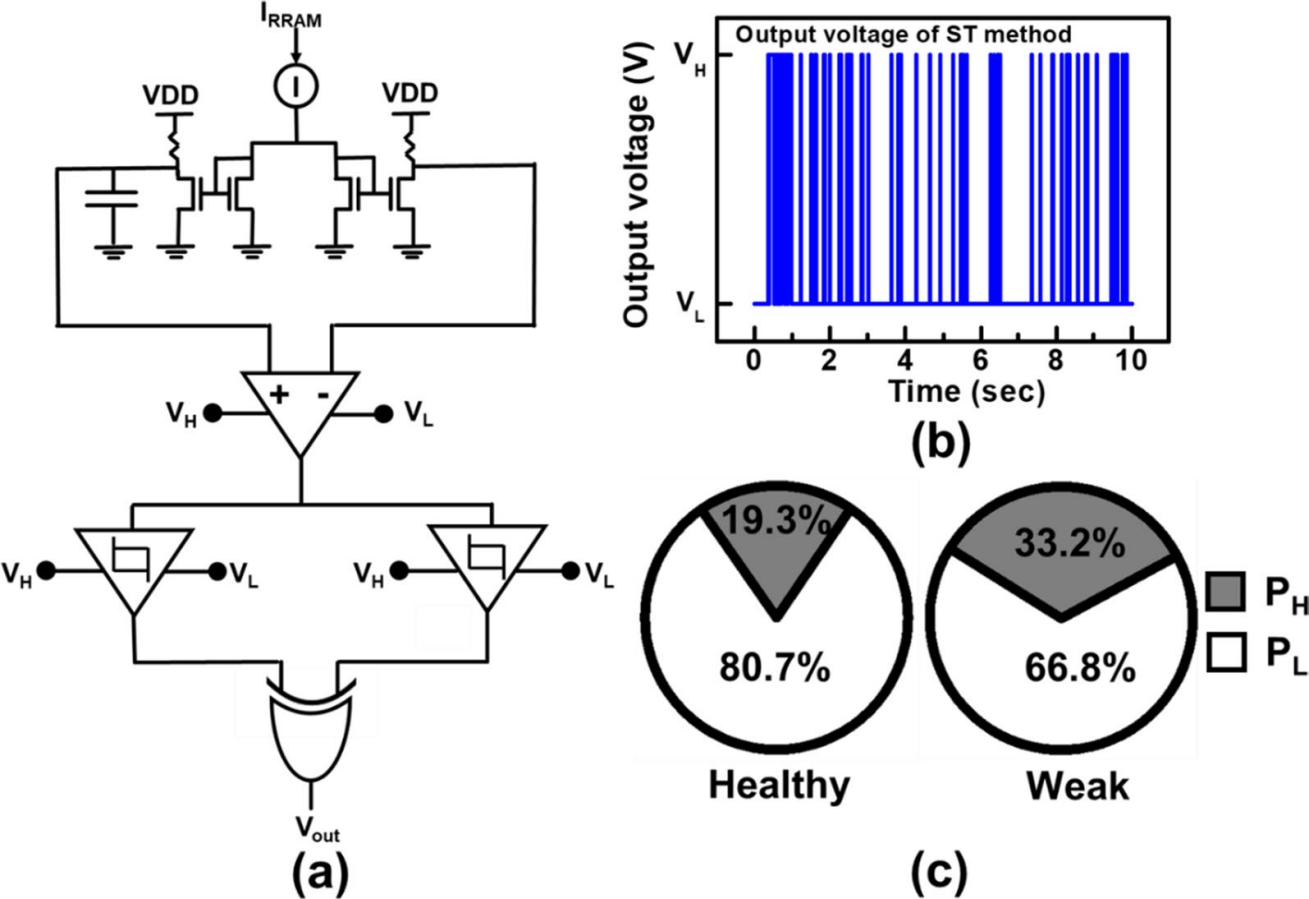
**a** Schematic of ST detection circuit and **b** its voltage output. **c** Pie charts of output voltages on healthy/weak cells in ST method. More VL can be obtained in a healthy cell, which is probably with only one dominant CF

The second circuit proposed here for screening out vulnerable cells, named as Schmitt trigger (ST) method, is illustrated in Fig. 9a. Two Schmitt triggers, which upper/lower triggers are designed to be 0.65 V/0.35 V and 0.55 V/0.45 V, respectively, are used instead to find out the probability of the read current in its middle state. Output voltage, shown in Fig. 9b, becomes high when the read current is in its middle state. From the ST method, the percentages of high/low levels on the XOR output are summarized in Fig. 9c. The detection outputs are more likely to stay in *V*_H_ for weak cells than that for healthy ones.

**Fig. 10:**
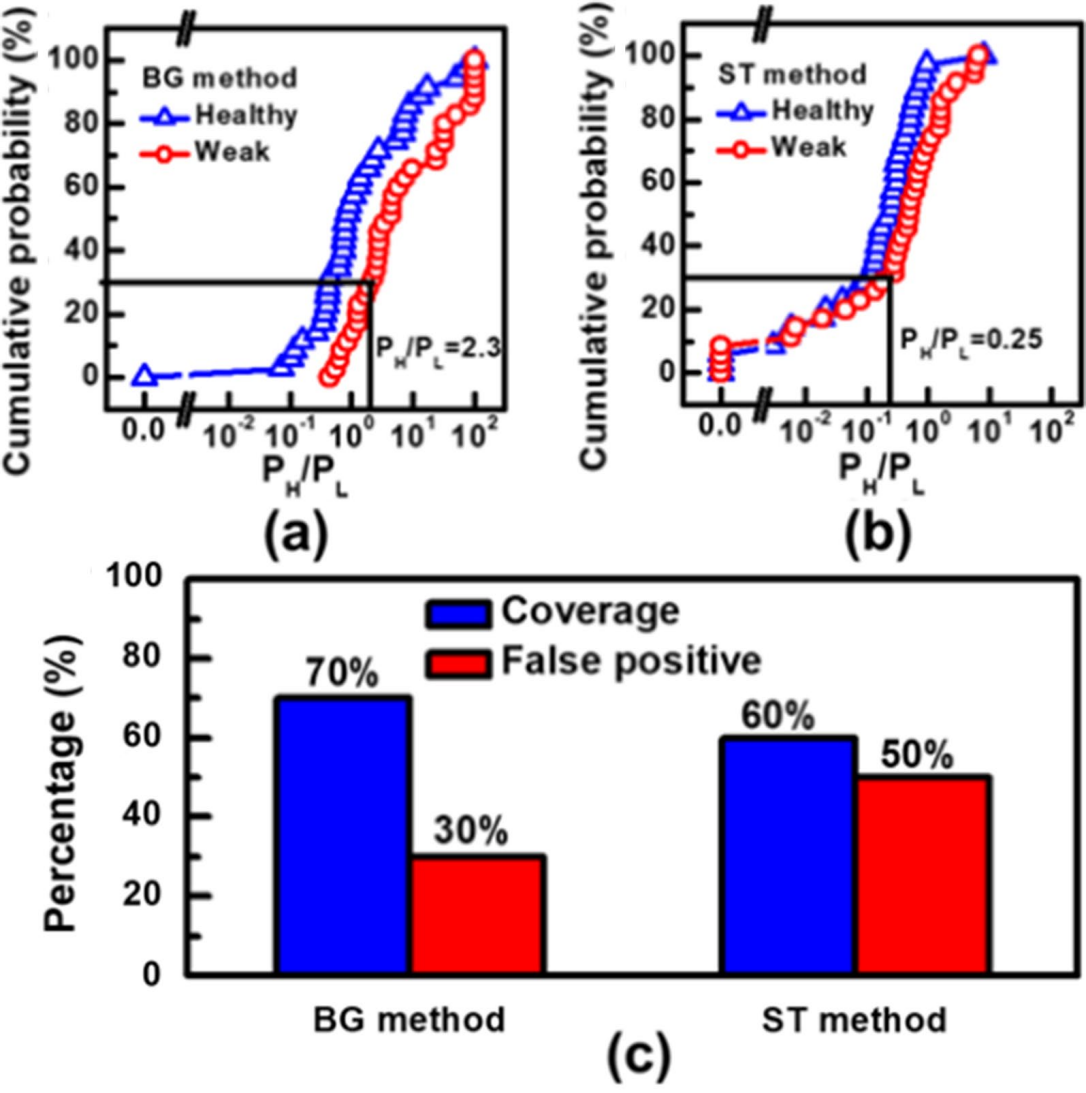
Cumulative distribution of ratio of probabilities in high/low logic states on different cells types in **a** BG method, **b** ST method. **c** Comparison of coverage rate and false positive rate between two circuit schemes

To investigate the success rate of the detection in identifying the weak cells, the ratio of high states from the detector output for the two groups of cells firstly categorized by LFN characteristics are compared in Fig. 10a, b. For the BG detection circuit, we defined a weak cell by having a *P*_H_/*P*_L_ ratio above 2.3. With this criterium, 70% of the weak cells can be successfully caught while leading to 30% false positive. For ST method, when the select criterium is set at a *P*_H_/*P*_L_ ratio > 0.25, the coverage rate can reach 60%, while the false positive can be as high as 50%. This makes ST method a less effective screen method. As compared in Fig. 10c, higher coverage rate and lower chances of false-positive are demonstrated by the BG method.

**Fig. 11:**
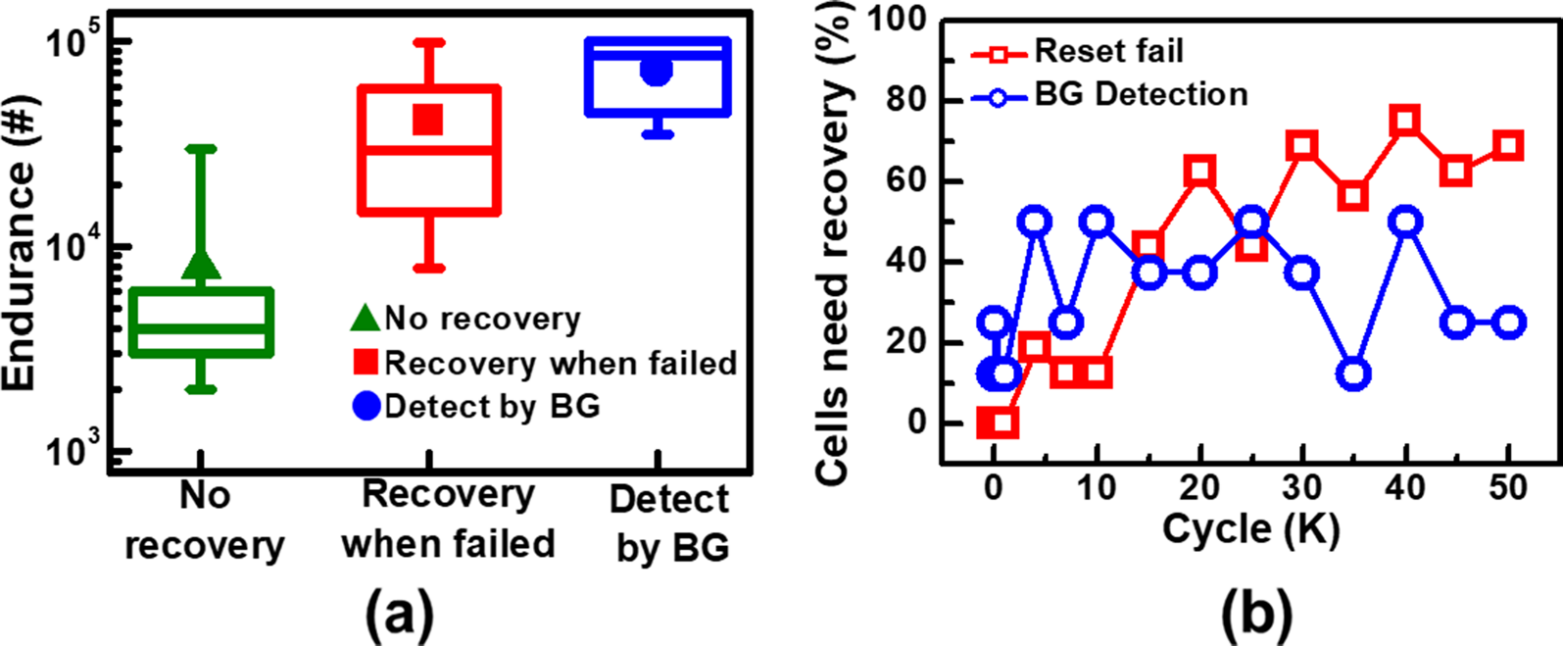
**a** Endurance comparison of different techniques, including recovery reset treatment and BG circuit detection. **b** Number of cells experienced recovery reset treatment during cycles

Due to high coverage rate provided by BG method, it is employed for the detection of vulnerable cells with high risk of endurance failure for the initiation of early interventions. The cycling characteristics of cells experiencing different types of recovery interventions are compared in Fig. 11a. Cells are found to sustain only 2 k cycles when no intervention is taken during cycling tests. Lifetime of BCRRAM can be extended by several thousand of cycles when reset recovery pulses are applied after reset failure. However, most of the revived cells cannot pass 8 k of cycling. Through the early detection circuit with BG scheme, weak cells in an array before cycling failure can be detected. With a recovery pulse applied on a weak cell detected, endurance of most BCRRAM cells can be significantly extended to more than 40 k cycles. The 15% cells in an 16 × 16 memory array required reset recovery treatment in different methods are compared in Fig. 11b. Although more cells need to be recovered before 10 k cycles in BG detection method, its percentage of cells is relatively stable throughout a 50 k cycling test. However, in comparison group, where devices are restored after reset failure, proportion of cells needed recovery interventions increases with cycling stress, which suggests heavier operation overhead on both speed and power.

Thanks to detection circuit and reset recovery, the cycling lifetime of BCRRAM can be extended effectively. Even though the coverage rate of the BG detection circuit reaches 70%, some vulnerable cells are not identified. As a result, we believe that improving the coverage rate is one of the pathways for further enhancing the overall endurance of BCRRAM arrays. The settings of the detection circuit can be further adjusted for decreasing the false negative rate, enhancing the coverage rate. Besides, the reset recovery reset can be optimized for better reviving BCRRAM to its cycling capabilities.

## Conclusions

In this study, correlations of LFNs, topographies of CFs and reset failure during cycling are established. Besides, a recovery reset treatment is implemented in BCRRAM array for restoring reset failure. Two detection circuits, BG method and ST method, are proposed and investigated to screen out the vulnerable cells for early recovery interventions. In addition, the proposed BG method with a higher coverage rate is employed on a BCRRAM array to improve endurance. With the newly proposed BG detection circuits and early detection initiated reset recovery operation, significant improvement of cycling endurance for over 10 k times has been demonstrated.

## Data Availability

The datasets supporting the conclusions of this article are included in the article.
